# Evidence-Informed Approach to De-Prescribing of Atypical Antipsychotics (AAP) in the Management of Behavioral Expressions (BE) in Advanced Neurocognitive Disorders (NCD): Results of a Retrospective Study

**DOI:** 10.3390/geriatrics7010014

**Published:** 2022-01-26

**Authors:** Atul Sunny Luthra, Raymond LinBin Gao, Shannon Remers, Peter Carducci, Joanna Sue

**Affiliations:** 1 Department of Psychiatry and Behavioural Neurosciences, McMaster University, Hamilton, ON L8M 1W9, Canada; 2 St. Peters Hospital, Hamilton, ON L8M 1W9, Canada; gaolin@HHSC.CA (R.L.G.); suejo@hhsc.ca (J.S.); 3 Homewood Health Inc., Guelph, ON N1E 6K9, Canada; sremers@homewoodhealth.com; 4 School of Pharmacy, University of Waterloo, Waterloo, ON N2G 1C5, Canada; pcarducc@uwaterloo.ca

**Keywords:** neurocognitive disorders, Behavior Expressions (BE), atypical antipsychotic medications (AAP), LuBAIR™ Paradigm and LuBAIR™ Inventory

## Abstract

The LuBAIR™ Paradigm is a novel approach to ascribe meaning to behavioral expressions in advanced neurocognitive disorders when the reliability of a clinical assessment is limited. The meaning ascribed to each behavioral category was used to identify those which are likely to respond to the use of atypical antipsychotics, in their management. De-prescribing was attempted on patients who qualified to enter this retrospective study. De-prescribing was defined as successful if individuals were completely withdrawn from AAP and remained off them for 60 days, without the re-emergence of behaviors. The LuBAIR™ Inventory was filled on two occasions. The data collected on the second occasion, in the successful and failed de-prescribed groups, were compared in this retrospective study. MANOVA, Chi-Square paired *t*-test statistical analyses were used to detect the differences in the behavioral categories between the two cohorts. Cohen d was used to measure effect size. Patients who did not have Mis-Identification and Goal-Directed Expressions were more likely to successfully de-prescribe: X2 (1, *N* = 40) = 29.119 *p* < 0.0001 and X2 (1, *N* = 40) = 32.374, *p* < 0.0001, respectively. Alternatively, the same behavioral categories were more likely to be present in patients who failed de-prescribing: MANOVA and paired *t*-test (*p* < 0.0001). Atypical antipsychotics, in their role as an antipsychotic and mood stabilizer, may be used to manage Mis-Identification and Goal-Directed Expressions, respectively.

## 1. Introduction

In accordance with the Diagnostic and Statistical Manual-5 [1], the diagnosis of behavioral symptoms in neurocognitive disorders (NCD) is one of exclusion. All mental illnesses (mood, anxiety psychotic disorders, and delirium), medical and environmental determinants for the presence of symptoms have to be identified, treated, and resolved prior to labeling them as ‘behavioral disturbances’ in Neuro-cognitive disorders (DSM-5). This framework to assess ‘behavioral disturbances’ in neurocognitive disorders has been adapted from the work done by Cohen-Mansfield (2003) who labeled it as agitation. Cohen-Mansfield further classified agitation into Verbally Aggressive and Verbally Non-aggressive and Physically Aggressive and Physically Non-aggressive [2]. The construct of agitation defined by Cohen-Mansfield is a clinical syndrome, in comparison to the term ‘agitation’ used in mental health literature, which describes a physiological state of ‘severe anxiety associated with motor restlessness’ [3]. Regrettably, the terminology of agitation has been used interchangeably, in clinical and research settings, with problematic interpretations [4,5,6]. One of the most used terminology to label symptoms in NCD is Behavioral and Psychological Symptoms of Dementia (BPSD) (DSM-IV-TR); defined as ‘agitation, apathy, depression, repetitive questioning, psychosis, aggression, sleep problems, wandering, and a variety of inappropriate behaviors’ [7]. The terminology of BPSD is without any specific criteria or definitions for each of these symptoms collected herein. Carrarini et al. (2021) [8] have used the terminology of ‘agitation’, defined as a ‘behavioral syndrome characterized by increased, often undirected, motor activity, restlessness, aggressiveness, and emotional distress. Cloak and Khalili (2020) [9] used the terminology of BPSD and defined it to include cognitive/perceptual (delusions and hallucinations), motor (pacing, wandering, repetitive movements, and physical aggression), verbal (yelling, calling out, repetitive speech, and verbal aggression), emotional (euphoria, depression, apathy, anxiety, and irritability) and vegetative (disturbances in sleep and appetite). Marcinkowska et al. (2020) [10] used the term BPSD and defined it to include ‘psychosis, agitation, aggression, depression, and anxiety’ and went on to focus on the therapeutic developments in the management of ‘dementia-related psychosis and agitation/aggression. As is evident, different terminologies have been used to describe the same constellation of symptoms and the same terms used with a different meaning in both clinical and research settings. Finally, the labeling of any terminology is from the standpoint of the observer rather than that of the person with NCD [2]. It is apparent that even the most basic definition of coining any terminology, defined as ‘a set of concepts and relationships that provide a common reference point for comparisons and aggregation of data’ [11], has yet to be consistently applied to the area of behaviors in advanced NCD.

Additionally, the reliability and validity of commonly used psychometric tools, in identifying individual clinical states or distinguishing amongst varied clinical states, in advanced stages of NCD is unknown [5]. According to the World Health Organization (2012) [12], the diagnosis of specific clinical states or distinguishing amongst different clinical states, in accordance with DSM, is increasingly difficult in advanced stages of NCD or in individuals with primary language abnormalities. This limitation in the diagnostic differentiation in advanced NCD is of huge clinical significance as ‘behavioral disturbances in advanced NCD’ is one of exclusion. It is also in the advanced stages of NCD when the prevalence of behavioral symptoms is upwards of 80% [13].

Additionally, the use of atypical antipsychotics (AAP) to manage these behaviors is without an understanding of their use as an antipsychotic [14,15,16], mood stabilizer [17,18], or as an augmentation of depressive or anxiety disorders [19,20]. A recent publication by McFarlane and Cummings (2021) [21] proposes that the minimal benefits of AAP may be due to their sedating properties.

This lack of consistency in the use of definitions on terminology to label behaviors as well as the absence of understanding of the indications for the use of AAP, have resulted in equivocal outcomes. There has been a demonstration of small but statistically significant benefits [22], no worsening after de-prescribing [23], and a degree of worsening after de-prescribing [24]. In many studies, placebo response rates were high and reflect a high rate of spontaneous resolution of these symptoms within three months [21]. Furthermore, the use of AAP do pose several risks from side effects in the form of excessive sedation and cognitive slowing, extrapyramidal symptoms, and gait disturbances with complications such as falls, orthostatic hypotension, and other cardiovascular complications and increased risk of death [10,25]. Consequently, it is recommended that individuals with NCD with BPSD be treated for a duration of three months for symptom stabilization, or a failed response to an adequate trial for three months, after which the AAP should be tapered and stopped [26]. The American Psychiatric Association (2016) [27] guidelines define de-prescribing as successful when the individual completely withdraws from AAP and remains off them for 60 days without the re-emergence of behaviors. Failed de-prescribing is defined as a relapse in behaviors early in the course of de-prescribing or failing to stay off AAP for a duration of 60 days without the re-emergence of behaviors. As is evident from the aforementioned arguments, the existing approach to labeling and treating behaviors in advanced NCD appears to offer limited potential for future growth.

An alternative approach to understanding the presence of behaviors in NCD has been gaining momentum since the conception of the Need-Driven dementia-compromised behavior model by Algase et al. (1996) [28]. Kovacs et al. (2005) [29] further developed this model. According to this model, instead of viewing behaviors as a problem, and from the observer’s point of view, the presence of behaviors is viewed as a manifestation of an unmet need. Therefore, behaviors are viewed as a mode of communication of this unmet need, and the meaning behind this behavior, that is the unmet need, requires decoding. The P.I.E.C.E.S.™ Learning and Development Model, [30] and Gentle Persuasive Approaches [31] offer guidance in ascribing the meaning behind the presence of behaviors in individuals with NCD and in the context of the ‘personhood’ of that individual. Whereas both approaches are viewed as huge steps forward in understanding the meaning of behaviors in individuals with advanced NCD, they fail to offer any specific framework to understand the meaning for the individual constellation or clustering of varied phenotypic manifestations of behavioral symptoms in advanced NCD. Ascribing the meaning to behavioral symptoms in the advanced stages of NCD through the traditional model of clinical assessment is particularly limiting as the reliability and validity of clinical examination under these circumstances is unreliable [32,33].

Adhering to the principles of defining the meaning of behavioral symptoms in advanced NCD, when clinical examination becomes un-reliable, the senior author published a book titled, ‘The meaning of behaviors in NCD: a Biopsychosocial model and classification of behaviors in NCD” [34]. The theoretical constructs in the book have formed the basis of the emergence of a philosophy of dementia care titled Luthra’s Behavioral Assessment and Intervention Response (LuBAIR™) Paradigm. The LuBAIR™ Paradigm is based on: a collection of ‘alike’ or similar’ behavioral symptoms sorted into individual behavioral categories; each behavioral category is adequately titled to represent the symptoms collected herein, reflects a specific meaning for the symptoms, and is justified by specific psychological theories which have been validated in the existing literature [34]. Table 1 outlines the various psychological theories, and the respective behavioral categories emanating from them, under the LuBAIR™ Paradigm. 

Individual behavioral categories in this classification system led to the development of a new dementia behavioral tool: Luthra’s Behavioral Assessment and Intervention Response (LuBAIR™) Inventory [35]. The LuBAIR™ Paradigm continues to evolve [35,36,37]. The LuBAIR™ Paradigm offers a framework for the generation of specific behavioral care plans for managing behavioral symptoms in advanced NCD [37]. Another proposed use of the LuBAIR™ Paradigm is to assist the prescribers in identifying behavioral categories that are likely to respond to the use of AAP in their management.

### Objective

The purpose of this retrospective chart review was to compare the behavioral categories identified in patients, on a specialized behavioral health unit, who were able to successfully withdraw from prescribed AAP with those behavioral categories in patients for whom de-prescribing failed. The study was approved by the Hamilton Integrated Research Ethics Board (#7202).

## 2. Materials and Methods

**Figure 1:**
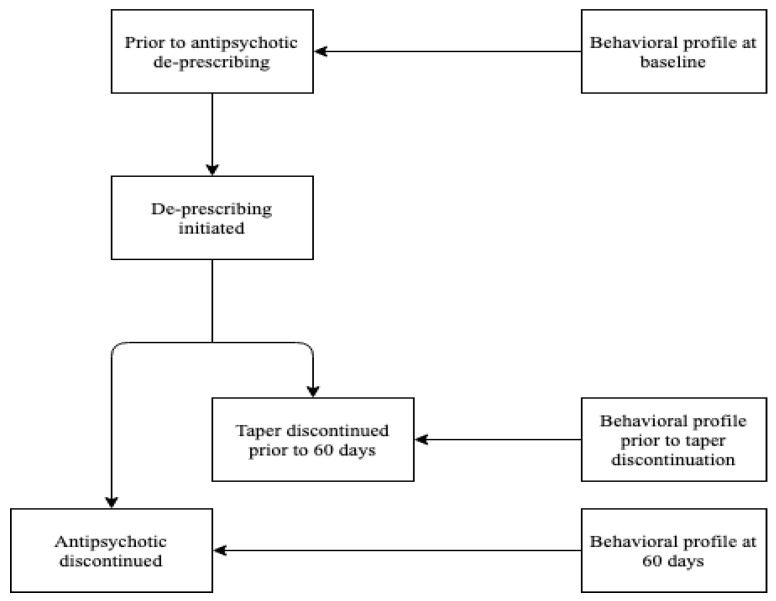
Schematic representation of the Care Pathway.

The study took place on a 63-bed Behavioral Health (BH) program in St. Peters Hospital, Hamilton, Ontario, Canada, that specializes in the assessment and management of behavioral expressions in advanced NCD, regardless of etiology. Once admitted to BH, a standardized care pathway is followed until discharge. The steps in this standardized care pathway are outlined in this paragraph. See Figure 1 for a schematic representation of the care pathway.

The referral diagnosis of advanced NCD for each patient admitted to the BH program is confirmed through a review of the history and a clinical examination. The LuBAIR™ Inventory is completed for each patient, for the first time, within two weeks of admission to BH by the attending geriatric psychiatrist in consultation with the assigned nurse case manager. To accomplish this, a review of the shift-by-shift descriptive behavioral charting by the nursing and interdisciplinary staff helps to establish the context in which the behavioral symptoms are occurring in order to make some assumptions about the meaning. The agreed-upon meaning is indicated on the LuBAIR™ Inventory. Behavioral care plans are developed using GPA™ principles and they are further modified in accordance with the meaning ascribed within the LuBAIR™ Paradigm and implemented daily. Pharmacological interventions, guided by the LuBAIR™ Paradigm, include attempting to de-prescribe AAP, partially or completely, in accordance with the APA guidelines, in all patients admitted to the BH program. In addition, there is the optimization of antidepressants along with the use of mood stabilizers (divalproex sodium and lithium). Pharmacological treatment also includes the use of ‘as necessary’ medications to manage high-risk situations. Each patient is reviewed at an inter-disciplinary clinical rounds meeting every eight weeks. A standardized clinical evaluation form is completed and based on the team discussion and a determination is made if there was a successful or failed de-prescribing. When the goals of the treatment are achieved (i.e., to ensure that there is mitigation of the risks associated with each behavioral category), the patient is then deemed ready for discharge to an alternate level of care. Due to the rolling nature of the admissions to BH, the patients are in varying stages of their journey. Patients admitted up to September of 2019 were selected as the study population. Patients were excluded from the study if they: (a) had a length of stay of fewer than 60 days; (b) had a history of mental illness requiring the use of AAP; and (c) were admitted without having been prescribed AAP. For patients who had successfully de-prescribed, clinical records were reviewed 60 days after the last dose of AAP. For the patients who failed de-prescribing, clinical records were reviewed to establish when the last dosage of AAP had to be increased or had to be re-introduced. The data from the most recent inter-disciplinary clinical rounds held closest to the respective dates was used to populate the LuBAIR™ Inventory for the second time.

### Statistical Analysis

Data was collected using the LuBAIR™ Inventory in order to detect differences in the behavioral categories between the two groups at the point of successful or failed de-prescribing as well as within groups between the first and the second data point. Three separate statistical tests were used to analyze these different data points. These tests included the MANOVA, chi-square, and paired *t*-test. When using MANOVA, an effort was made to cross factor the time of observation with behaviors in each of the behavioral categories. The purpose of doing this was to establish the relationship between individual behavioral categories and one or both of the variables of de-prescribing and time of observation. The Pillai test of fit was applied to the model. A chi-square test of independence was performed to examine the relationship between individual behavioral categories and successful or failed de-prescribing. Finally, the paired *t*-test was also used to examine the relationship between the behavioral categories on the LuBAIR™ Inventory done on the first and the second occasions, after attempted de-prescribing. To reduce the risk of Type 1 error, a *p*-value of <0.005 was considered statistically significant [38,39]. Clinical effect size was also measured using Cohen’s d, 0.2 (small effect), 0.5 (moderate effect), and 0.8 (large effect) [39]. R was used to conduct the data analysis.

## 3. Results

Forty (40) patients qualified to enter the study. Table 2 provides the individual demographics and behavioral categories in the successful de-prescribed group.

Seventeen (17) patients were successfully de-prescribed off AAP. Table 3 provides individual demographics and behavioral categories in the failed de-prescribed group. Twenty-three (23) patients were in the failed de-prescribed group.

Table 4 provides information on the behavioral frequencies of the patients who qualified to enter the study (*N* = 40). 

Table 5 provides data on the MANOVA analysis and the cross-factoring of the time and de-prescribing results with the behavioral scores. The MONOVA results found that both de-prescribing and time of observation were significant when cross-factored against behavioral categories. When a summary of the individual behavioral categories was examined, four behavioral categories were found to be significant. Mis-identification and goal-directed expressions were found to have a statistically significant correlation with both time and de-prescribing (mis-identification expressions; time < 0.0001 and de-prescribing < 0.0001 and goal-directed expressions: time < 0.0001 and de-prescribing < 0.001). Vocal expressions were found to have a statistically significant correlation only with time (<0.0047) but not with de-prescribing (*p* = 0.1560). Motor expressions were found to have a statistically significant correlation with de-prescribing (*p* = 0.01107) but not with time (*p* = 0.1680).

Table 6 provides the data of the chi-square test of independence on the relationship between behavior categories and successful de-prescribing.

Patients who did not have the behavioral category of mis-identification expressions were more likely to successfully de-prescribe, X2 (1, *N* = 40) = 29.119 *p* < 0.0001. Additionally, patients who did not have the behavioral category of goal-directed expressions, X2 (1, *N* = 40) = 32.374, *p* < 0.0001, were also more likely to successfully de-prescribe. Vocal expressions did not reach statistical significance in the chi-square test, X2 (1, *N* = 40) =1.7684, *p* = 0.184. The remaining behavioral categories did not reach statistically significant results on chi-square test (emotional expressions; *p* = 0.2638, fretful-trepidated expressions; *p* = 0.000, importuning expressions; *p* = 0.0093, apathy expressions; *p* = 0.000, physical expressions; and *p* = 0.0746, and sexual expressions; *p* = 0.000). Table 7 provides data on the behavioral categories on the first and the last assessment (paired T-Test *N* = 40). Patients who had mis-identification or goal-directed expressions showed statistically significant results (*p* < 0.0001 and *p* < 0.0001, respectively) with moderate to large changes skewing towards the positive direction (Cohen d value of 0.9874 and 1.0381, respectively). These findings suggest that these two behavioral categories were present in the patients who failed de-prescribing or had an increase in these behavioral symptoms if the medications were to be removed. Vocal expressions also showed statistically significant results at a lower level of power (*p* = 0.0047) and skewing in the positive direction (Cohen-d value of 0.6081), thereby suggesting a potential increase in this category of expressions with de-prescribing. The behavioral categories that did not reach statistically significant results on the paired *t*-test include emotional expression; *p* = 0.1599, fretful-trepidated expressions; *p* = 0.4865, importuning expressions; *p* = 0.4865, apathy expressions; *p* = 0.3235, oppositional expressions; *p* = 0.3235, physical expressions; *p* = 0.5703, and sexual expressions; *p* = 0.1599. In summary, only two of the behavioral categories of mis-identification and goal-directed expressions reached statistically significant results on all three of the tests applied to the data, thereby suggesting an increase in these categories upon de-prescribing or an increased likelihood of their absence in the successful de-prescribed group. Vocal expressions showed significant results on the time of observation but not the de-prescribing variable on MANOVA, failed to show significant results on chi-square, and did show a statistically significant result, at lower power, on the paired *t*-test. Motor expressions showed significant results on de-prescribing but not on the time of observation variable on the MANOVA.

## 4. Discussion

Preliminary results suggest that two behavioral categories in the LuBAIR™ Paradigm, (a) Mis-identification Expressions (MiE) and (b) goal-directed expressions may justify the use of AAP as a part of a comprehensive behavioral care plan. The reasons for equivocal results for the behavioral category of vocal expressions are described in the following paragraphs of the ‘discussion’ section. Motor expressions, also commonly referred to as ‘agitation’, are the most ubiquitous non-cognitive symptom presentation in advanced NCD, yet the most non-specific in its interpretation. Under the LuBAIR™ Paradigm, the purpose for the presence of motor expressions is simply to highlight a state of unrest in an individual with advanced NCD but has no meaning for its presence. The only way to ascribe meaning to their presence is through its affiliation with one of the other behavioral categories, since motor expressions always occur in conjunction with one or more of the other behavioral categories in the LuBAIR™ Inventory. Hence, it is expected that the motor expressions will surface in all the patients with failed de-prescribing of AAP. Therefore, the only way to manage motor expressions is to successfully manage, individually or with clustering, the behavioral categories with them in accordance with the guidance offered by the LuBAIR™ Paradigm. 

### 4.1. Use of Atypical Antipsychotics for Mis-Identification Expressions

The use of AAP in the management of Mis-identification Expressions (MiE) may be justified because of the specific psychological construct used to support the existence of this specific behavioral category, which is based on the impairment of information processing circuits, because of the dementia process [40]. Atkinson and Shiffrin (1968) [41] proposed a model for the cascade of the flow of information in the brain, along the information processing pathways, and governed by the Theories of Information Processing. The emergence of MiE occurs due to impairment in the information processing pathways in the moderate to advanced stage of dementia [40,41]. The impairment of the information processing pathways specifically occurs at the level of encoding, the second step in the formation of short-term memories [30,41,42]. The specific disconnect occurs at the level of the paring of the final two processes involved in encoding: pattern recognition and schema identification [43]. The primary purpose of these two processes is to pair the new information with the stored information to provide context and meaning for the newly received information. This incorrect pairing of the new information with the stored information leads to an altered sense of relatedness, and reality, between self and the milieu. The stored information, to which the new information is paired in any given set of circumstances, is always of profound emotional importance to the individual with moderate to advanced NCD. Consequently, there is an impaired pairing of the incoming visual (person, places, and objects), auditory, gustatory, olfactory, and tactile stimuli, to stored information, thereby leading to the misidentification of the incoming stimuli [44,45]. Likewise, information emanating from real-time ‘events and occurrences’ will be incorrectly paired with stored experiences, thereby leading to misconstruing of the incoming stimuli, which will result in an altered sense of the reality of that experience. Finally, the information arising from interpersonal interactions between the individual with advanced NCD and the staff or family members will be incorrectly paired with the stored information, thereby leading to misinterpretation of the interaction, which will result in an altered sense of reality. Hence, the impairment in the encoding step in all those aforementioned steps leads to an altered sense of relatedness, and reality, between self and the milieu for the individual with advanced NCD. Psychosis is defined as a breakdown in reality for an individual. Therefore, the meaning ascribed to the behavioral category of MiE is, “Please help me- my thinking is not based in reality”. Psychosis is defined as a breakdown in reality for an individual. The purpose for the existence of this behavioral category is that it represents an alternative approach to the identification of psychosis in individuals with moderate to advanced NCD, when it is challenging to obtain a reliable history and conduct a valid mental state examination [46]. The reason for the success of the use of AAP for this behavioral category is due to its ability to treat psychosis in this cohort of the population. Hence, the indication for use of AAP to manage the behavioral symptoms in this behavioral category is as an antipsychotic.

### 4.2. Use of Atypical Antipsychotics for Goal-Directed Expressions

The use of AAP in the management of Goal-Directed Expression (GDE) may be justified because of the specific psychological construct used to support the existence of this behavioral category which is based on the impairment of the motivational circuits due to the dementia process, resulting in heightened motivational drives.

Motivational theories [29,47] govern the understanding of the functioning of the motivational circuits. A discrepancy between an individual’s internal physiological or psychological state and the external environment leads to the identification of a need, which in turn is represented as cognitions [48]. Cognitions lead to the formation of goals, which in turn, triggers ‘drives’ or ‘motivation’. It is the motivational drive, which propel goals to completion thereby satiating the identified need. This leads to the release of the need with the re-establishment of homeostasis [49]. In dementia of different etiologies, and at varying stages of the disease, impairment in motivational circuits can result in the generation of heightened motivational drives. This leads to a heightened detection of the discrepancy between the individual’s physiological or psychological state and its environment, thereby resulting in the identification of several needs, simultaneously or sequentially [47]. An attempt to propel all the identified needs, represented as cognitions and goals, to completion results in the manifestation of a behavioral expression that appears persistent, repetitive, and determined. The aforementioned cascade of steps leads to the clinical presentation of ‘high energy’ or busy beaver states. The need requiring satiation in this behavioral category involves either belongingness or innate physiological needs. The meaning expressed in this behavioral category is “Please help me belong…” [50,51] or “Please help me satiate my innate physiological needs…” [51]. The belongingness need includes ‘family, faith, community or ethnicity, and professional organizations or life’s work’ [50,51] One example of the behaviors under this category include, an individual incessantly wanting to go to the bank to pay their dues due to the fear of losing their house if they forfeited on their mortgage payment. This behavior is driven to satiate the need for belongingness to the ‘family’ as a bread-winner’ and a ‘provider’. Another example of the behaviors under this category includes, an individual repeatedly calling family members to take them to a church service and refusing to take “no” for an answer. This behavior is driven to satiate the need for belongingness to their ‘church’ and their ‘faith’. Yet, another example of behaviors under this category includes an individual directing other residents on the unit to get their job done, yelling at them to stop being lazy and if they did not get their work done in time they would be fired. This behavior is driven to satiate the need for belongingness to their ‘life’s work’, in which they took immense pride. Their innate physiological needs include hunger/thirst, fatigue/need to rest, voiding/defecating, pain or discomfort/need for relief, mental and social stimulation, and the need for intimacy and pleasure including sexual needs [51]. Examples of behaviors under this category include persistent requests to be toileted, constantly calling for the nurses to take them to bed while they are in bed, and sustained ringing of the call bell to complain of pain and requesting the nurse for medications. In each of these examples, the need requiring satiating may be the actual physiological need or more importantly the need for ‘mental and social stimulation’. Each of these behavioral manifestations persists for hours on end, shift after shift, and are not amenable to interpersonal or environmental interventions. Goal-directed behaviors are a cardinal and core symptom presentation in manic and psychotic syndromes, regardless of the etiology [52,53]. In manic episodes, the heightened motivational drives to satiate various needs are often determined by virtue of what is emotionally important to that individual at that given point in time [54]. As an example, is it going to be the drive to satiate pleasurable needs or self-actualization and establishing legacy needs which will be determined by the discrepancy between the individual’s internal physiological and/or psychological state and their environment. It is this pathophysiology of the illness, regardless of the etiology, which responds to mood stabilizers of the types of divalproex, lithium, and recent literature’s addition of AAP to the category of mood stabilizer. Likewise, in psychotic episodes, the intensity of the fixation on the falsehood of the cognitions will determine the extent to which the individual will drive the goals to completion [55,56]. As an example, the intensity of fixation on the falsehood of the cognition that there is a transmitter embedded in the television will determine the intensity with which they will establish the goal to dismantle the television, to find the transmitter. In all these aforementioned clinical states, the tenacity with which the goals are established determines the severity of generation of the motivational drives; and they are always high. The presence of symptoms of GDE in advanced neurocognitive disorders likely represents a collection of symptoms in a syndrome which is yet to be characterized and needs further study. A syndrome is a constellation of recognizable traits or symptoms which tend to group together, run together, and/or respond to a common therapeutic modality [34], regardless of the etiology. Based on these principles, it is being hypothesized that some of the manifestations of the symptoms under the behavioral category of GDE respond to the mood stabilization effects of AAP, while the others respond to the antipsychotic effects of AAP.

### 4.3. Equivocal Response of Vocal Expressions to AAP

The response of the behavioral category of vocal expressions to AAP failed to reach statistically significant results in both of the statistical tests applied to the data. The reason for this lies in the heterogeneity of the clustering of the clinical symptoms under this behavioral category. Under the LuBAIR™ Paradigm, there are six subtypes of vocal expressions and each subtype is based on its own psychological construct which justifies its presence [34,35,36,37]. The majority of clinical symptoms aggregated under this behavioral category are based on dysregulation of the primary emotions of anger and joy. The expressions of the symptoms, which are based on the dysregulation of primary emotions of anger, are represented on a continuum from lowest to the highest severity. The lower severity may take the form of ‘brief explosive outburst’ (the first example in the first bullet under Vocal Expressions in Table 1) or ‘talking loud, incessantly, and fast’ (the first example in the second bullet under Vocal Expressions in Table 1). In the adult mental health literature, the former example is usually a manifestation in both depressive [57] or ‘dysphoric’ episodes [58] and requires the use of anti-depressants or AAP to respond, respectively. As the intensity of dysregulation of primary emotions of anger further escalates, it leads to the manifestation of dysphoric syndromes [58], quarrelsome and destructive syndromes [53,59] (second example in the first bullet under Vocal Expressions in Table 1). Continued escalation in the dysregulation of primary emotions of anger may ultimately result in ‘screaming or noise-making behaviors’ [60] (last bullet under Vocal Expressions in Table 1). All of these manifestations are usually observed in manic or psychotic episodes in the adult mental health literature, and therefore likely to respond to AAP, both in its indications as a mood stabilizer and antipsychotic, respectively. The symptoms based on the dysregulation of the primary emotion of joy are manifested as ‘manic-like behaviors’ [53] (second example in the second bullet under Vocal Expressions). The example in the third bullet under Vocal Expressions (Table 1), ‘yelling and screaming to get things done’, is an example of ‘goal-directed cognitions’; a step preceding the emergence of goal-directed behaviors. The rationale for its response to AAP has been described above. The first example in the fourth bullet under Vocal Expression includes ‘rattling bed rails/tabletops’ is an example of importuning expressions. The creation of the behavioral category of importuning expression is based on the drive to satiate ‘innate physiological needs’ and can either be the manifestation of depressive episodes [61] or that of manic episodes in the form of heightened seeking of pleasurable needs [53]. Importuning as a symptom of depressive episodes will respond to an antidepressant and as a manifestation of the manic episode will respond to a mood stabilizer including AAP. Finally, the second example in the fourth bullet under the Vocal Expressions is ‘calling out for family/friends and is an example of fretful expressions. The creation of the behavioral category of fretful-trepidated expressions is based on the drive to satiate security needs, due to impairment in the regulation of primary emotions of fear. Emotions and cognitive schema of fear are present in anxiety and depressive syndromes [62] in the adult mental health literature and respond to the use of anti-depressants. As is evident, there is a vast heterogeneity of the symptoms collected under the behavioral category of vocal expressions, each being supported by a different psychological construct for their existence. In this retrospective study, no attempt was made to stratify the patients into the formation of a pure sample of vocal expressions and defined by similar psychological constructs, and instead, they were all clumped together and their response to AAP was assessed as a group effect. In the patients who were recruited for this retrospective study, there appears to be a preponderance of the symptoms, under the vocal expressions, which had the propensity to respond to AAP, but the cohort was not pure enough nor were the numbers high enough, to reach statistically significant proportion. Further studies are in order to stratify individual symptoms under vocal expression, in accordance with each of the psychological constructs used to justify their existence, in order to form a pure sample, and the pharmacological treatment tailored according to it, to determine their responsivity.

### 4.4. Absence of Response of Dis-Organized Expression to AAP

Under the LuBAIR™ Paradigm, the behavioral category of Dis-organized Expressions is based on impairment in the regulation of sensorium, thereby resulting in abnormalities in the level of alertness and attention. Impairment in alertness results in the slowing down of information processing speed and delayed reaction time and the impairment in attention impacts on the ‘chunk’ of information being inputted onto the information processing pathways. The impairment in the combination of the two above steps results in reduced task performance, thereby untowardly affecting the intellectual, emotional, and psychological functions and functional decline. The presence of dis-organized expressions is due to the alteration in body physiology due to illness, caused by any etiology. The indiscriminate use of psychotropic medications, including AAP, would be responsible for the causation of dis-organized expressions and would resolve with their removal. The cohort of patients included in this retrospective study did not exhibit any dis-organized expressions. Had they been present at the time of initial evaluation, they would have only resolved with the removal of the AAP, and quite the opposite of what was observed in the case of the two behavioral categories of mis-identification and goal-directed expressions.

### 4.5. Absence of Response of Emotional and Fretful Expressions to AAP

Under the LuBAIR™ Paradigm, the behavioral categories of Emotional and Fretful Expressions are based on the impairment in the regulation of the primary emotions of discontentment and melancholy and fear, respectively. In the adult mental health literature, the presence of cognitive schema and emotions of discontent (negativity), melancholy, and fear are often symptom manifestations in anxiety and depressive episodes [62,63] and require the use of antidepressants to manage them. This would explain why these symptoms, under the behavioral category of vocal expressions, may have been minimally affected by the presence or removal of AAP.

### 4.6. Absence of Response of Apathy Expressions to AAP

Under the LuBAIR™ Paradigm, the behavioral category of Apathy Expressions is based on the absence of the ability of the individual to detect the discrepancy between the internal psychological and/or physiological state and that of their milieu, thereby lacking the ability to recognize a need. The absence of the recognition of a need results in the absence of formation of a goal, whereby there is no triggering of motivational forces. In adult mental health literature, these a-motivational states are usually symptom manifestations of functional mental illnesses like depressive episodes [64] or catatonic states [65] and may require antidepressants, stimulants, or lorazepam to manage them, depending upon the clinical conditions. For these reasons, symptoms under the behavioral category of apathy expressions may have been minimally affected by the presence or removal of AAP.

### 4.7. Absence of Response of Oppositional Expressions to AAP

Under the LuBAIR™ Paradigm, the behavioral category of Oppositional Expressions is based on the psychological constructs used to define ‘compliance’ in developmental psychology [66] and the only way to manage non-compliance is through behavioral care planning, as no pharmacological interventions have been shown to be effective. Hence, the presence or absence of AAP would have minimal impact on the symptoms under the behavioral category of oppositional expressions.

### 4.8. Absence of Response of Physical Expressions to AAP

Under the LuBAIR™ Paradigm, the emergence of symptoms in this behavioral category is due to the perceived impediment, by the individual with advanced NCD, of goal attainment with subsequent generation of emotions based on discontentment and anger [67]. Since the established goal will vary in a different set of circumstances, so will the perceived impediment of satiation of these goals. For example, the emergence of symptoms of physical expressions may be due to the perceived impediment of satiation of innate physiological needs (as is the case during the presence of importuning expressions), security needs (as is the case during the presence of fretful expressions), or belongingness needs (as is the case during the presence of goal-directed expressions). Another example for the emergence of physical expressions may be due to the perceived imposition of the caregiver’s needs on to the individual with NCD, due to the individual’s inability to recognize their own need for ‘care of self or milieu’, as is the case in apathy expressions. Likewise, there may be the emergence of physical expressions due to perceived impediment of attainment of the goal of avoiding pain, as is the case of manifestation of emotional expressions based on emotions of discontentment. Alternatively, there may be the emergence of physical expressions due to perceived impediment of attainment of the goal of coping with pain, as is the case of manifestation of emotional expressions based on emotions of melancholy. Finally, there may be the emergence of physical expressions due to perceived impediment of the attainment of the goal of caregivers heeding to their defensive responses, as is the case of manifestation of vocal expressions based in anger. As is evident, the causes of physical expressions are determined by which particular goal attainment is being impeded in a given set of circumstances. Theoretically, we should have seen the behavioral category of physical expressions reach statistically significant proportions in the failed de-prescribed group. It can be hypothesized that the removal of AAP and staying off AAP was not allowed for a long enough time, in order for the mis-identification and goal-directed expressions to escalate, whereby the needs associated with them were impeded for the emergence of physical expressions. It is also possible that the behavioral care plan put in place for these specific behavioral categories, again in accordance with the LuBAIR™ Paradigm, was successful in mitigating the behavioral risks such that it did not escalate to the emergence of physical expressions. Alternatively, since there was always a clustering of behavioral expressions in each case, there would have been several needs requiring satiation and just the perceived impediment of satiation of the needs associated with either mis-identification or goal-directed expressions would not have been sufficient to generate physical expressions.

Sexual expressions were conspicuously absent from this cohort of patients recruited for this retrospective study. However, in accordance with the LuBAIR™ Paradigm, there is also a vast heterogeneity in the behavioral category of sexual expressions. There are six subtypes of sexual expressions, each based on a specific psychological construct supporting them. Sexual expressions based on the misidentification of facial visual stimuli, thereby resulting in over-identifying another female resident as their spouse (mis-identification expressions) or sexual expressions based in ‘attribution error’. The latter is based on the mislabeling of behavioral symptoms as a sexual expression when upon re-review of the situation, it was not. Sexual expressions based in ‘stimulus bound tendencies’ which are based on the impairment of self-regulatory and monitoring circuits form another subtype. Then there are sexual expressions based on the satiation of innate physiological needs of sexuality due to heightened motivational drives (goal-directed expressions) or those based on the satiation of innate physiological needs of social and mental stimulation (importuning expressions), often referred to as intimacy needs in dementia literature [68]. The final subtype of sexual expressions are those based on the satiation of security needs, presenting as ‘clingy’ or ‘latching on’ behavioral symptoms (fretful expressions). Based upon the results of this retrospective study, sexual expressions based on mis-identification and goal-directedness have a likelihood of responding to AAP.

Another variable, which requires discussion in the context of this retrospective study, is if there is any relationship between the dose of use of AAP in managing behavioral expressions and their likelihood of being successfully de-prescribed. Gao et al. [69] published a study exploring this relationship and found that there was a threshold for each of the three AAP: seroquel at a dose higher than 50 mg, olanzapine at a dose higher than 1.75 mg, and risperidone at a dose higher than 0.5 mg were associated with worse outcomes upon discontinuation of these medications. All of the patients in this cohort of the retrospective study were on doses higher than the ones identified in the study by Gao et al. [69], both in the failed and the successful de-prescribed groups. If the dose of AAP in each patient was to be the confounding variable for the outcome of this study, we would not have had any successful de-prescribed patients at all.

### 4.9. Limitations of the Study

There are several limitations of this retrospective study. Completion of the LuBAIR™ Inventory using existing clinical records, which used terminologies such as ‘agitation’ and ‘aggression’ to label behaviors, was problematic. Unless there were good clinical descriptors in allied health staff documentation, the risk of using inaccurate information to complete the LuBAIR™ Inventory was high. The second limitation of the study is the relatively small sample size. However, to compensate for this obvious limitation, two different statistical tests were used to evaluate the data, one of which is able to identify the effect size of the results. Pearson’s chi-square statistical test was chosen as it is extremely sensitive to sample size [49]. A strong association may not be demonstrated if the sample size is small and even trivial associations are delineated by increasing the sample size. The fact such a small sample size has demonstrated statistically significant differences between the two groups provides a high level of credibility to the presence of these differences under the null hypothesis. Secondly, the results of the paired *t*-test were adjusted to include the ‘size effect’, using Cohen’s d test. The results revealed statistically significant results with a large effect size for the presence of Mis-identification Expressions (MiE) and Goal-Directed Expressions (GDE) in the failed de-prescribing cohort, thereby further enhancing the credibility of the results of this retrospective study.

## 5. Conclusions

The use of the LuBAIR™ Inventory and Paradigm may have the potential to predict which behavioral categories associated with advanced NCD may justify the use of AAP in their management. In conjunction with a comprehensive behavioral care plan, there may be justification for the use of AAP in the management of only two behavioral categories: Mis-Identification Expressions (MiE) and Goal-Directed Expressions (GDE), in their role as an antipsychotic and mood stabilizer, respectively. There are subtypes of vocal expressions which may justify the use of AAP in their management, but this category requires further study. The results of this study may be used to justify de-prescribing of AAP in their use for all the remaining behavioral categories in the LuBAIR™ Inventory. Furthermore, the results of this study are a preliminary step towards offering evidence to support the psychological constructs used to posit the meaning of behavioral expressions, under the LuBAIR™ Paradigm. Finally, the results of this study will be used to generate a priori hypotheses to develop a longitudinal prospective study to validate the use of AAP in the management of certain behavioral categories under the LuBAIR™ Paradigm.

## Figures and Tables

**Table 1:** Psychological Constructs, Behavioral Categories emanating from them and individual symptoms collected herein, under each Behavioral Category. This is an adaptation of the LuBAIR™ Inventory. Available on line: http://www.dementiabehaviors.com (accessed on 11 December 2021).

Impairment in Regulation of Sensorium (Based in Information Processing Theories)	Impairment in Emotional Regulatory Circuits (Based in Theories on Regulation of Emotions)
**Disorganized Expressions (DOE)**	**Vocal Expressions (VE)**
Appearing “vacant” or “lost” in facial expressions Disorganized thinking, unintelligible or gabled speech Rapid shifts in, or incongruence of, emotional states Inappropriate mixing of food or dressing, layering of clothes, smearing fecal matter, playing in the toilet bowl, etc. Playing with “things” in the air, responding to auditory hallucination & picking “things” from the body or furniture. Mental or physical lethargy , or general functional decline	Verbally responsive (brief explosive burst, argumentative, quarrelsome) Talking loud and fast, manic-like behavior Yelling and screaming to get things done Rattling bed rails/table tops, persistently calling out for staff/family member Uttering noises or making repetitive sounds
	**Emotional Expressions (EE)**
	Appearing sad, tearful or irritable Expressing themes of despair, morbidity, somatic complaints or self-deprecating comments Mimicking/mocking or being dismissive Sarcastic or teasing, being derogatory, critical and negative of others Expressing feelings or rejection or increased sensitivity to others comments
**Impairment in Information Processing Pathways** **(Based in Information Processing Theories)**	**Fretful-Trepidated Expressions (FE)**
	Expressing worry, fear, forbidding, or catastrophe
**Mis-Identification Expressions (MiE)**	Fearful or scared facial expressions
Misidentification of persons, places, objects Misidentification of sounds, smells, tastes, or touch Misidentification of events, or occurrences Misperception or misinterpretation of comments or behaviors of others	Anxious or distressed facial expressions Clingy or “latching on”, wringing of hands, rubbing face/body Hoarding or collecting
**Impairment in Motivational circuits (Based on Motivational Theories)**	**Impairment in Self-Monitoring and Regulatory Circuits. (Based in Theories on Regulation of Social Behaviors)**
**Goal-Directed Expressions (GDE)**	**Oppositional Expressions (OE)**
Goal-directed thinking: I am going home to my kids/to the bank; I am getting married to day; where can I pay my bills Goal-directed activities: rummaging, hoarding, empting drawers etc. stripping of clothes, rearranging furniture or fixing items in melleu bed/chair exiting or exit seeking; intrusiveness or purposeful wandering	Negotiating around care and other needs Working against the care provider (pulling pants up during incontinent care, buttoning up shirt when needing to undress) Evasive to directions from care provider (ignoring etc.) Resistive to care, medications, or meals (pursing lips at meals, clamping/crossing legs, closed, tightening arms, resisting rolling/turning etc.) Barricading and territorialism of space or belongings
	**Physically Responsive Expressions (PE)**
	Self-abusive Pulling, pushing, grabbing Kicking, biting, scratching, punching, twisting, head-butting, spitting at someone Throwing things, breaking objects, tipping furniture
**Apathy Expressions (AE)**	
Indifference and/or lack of concern re-self and environment Lack of self-initiation, low social engagement (interpersonal interactions and milieu structure, poor resistance Emotional indifference and/or lack of emotional remorse	
**Importuning Expressions (IE)**	

Persistently seeking reassurance or asking for assistance Behaving in ways for demands to be met immediately (repeatedly asking to be toileted or for medications, etc.) Shadowing staff (following closely, crowding staff member’s personal space) Attention seeking or “manipulative” behaviors (repeatedly empting soiled line cart, throwing food on the floor, etc.)	**Sexual Expressions (SE)**
	Verbally sexual (comments, gestures, innuendos) Physically sexual (grabbing breasts, buttocks) Self-stimulation
**Motor expressions (ME)**	
Roaming, pacing, wandering Fidgety, pocking in chair, restless, agitated Seemingly driven, “on the go”, wheelchair propelling, chair/bed exiting	

**Table 2:** Individual Demographics and Behavioral Categories in the Successful de-prescribed group. Columns show the individual patient’s demographics of identification number, age, and the initial and the second date of data collection. Rows show the individual behavioral categories with 0 representing the absence of the category and 1 representing the presence of the category.

Patient Number	Age	Date	Behavioural Categories											
			Disorganized Expressions	Mis-Identification Expressions	Goal-Directed Express-ions	Vocal Expressions	Emotional Expressions	Fretful-Trepidated Expressions	Importuning Expressions	Apathy Expressions	Oppositional Expressions	Physical Expressions	Sexual Expressions	Motor Expressions
001S	Age: 76M	31/12/2018	0	0	0	1	1	0	0	0	1	1	0	1
		8/1/2019	0	0	0	1	1	1	1	0	1	1	0	1
002S	Age: 96M	27/06/2019	0	0	0	1	1	1	1	0	1	1	0	1
		15/09/2019	0	0	0	1	1	1	1	0	1	1	0	1
003S	Age: 87F	20/06/2018	0	0	0	1	1	0	0	0	1	1	0	0
		12/8/2018	0	0	0	1	1	1	1	0	1	1	0	1
004S	Age: 70M	5/7/2019	0	0	0	1	1	1	1	0	1	0	0	1
		11/10/2019	0	0	0	1	1	1	1	0	1	1	0	1
005S	Age: 81 M	23/03/2019	0	0	0	0	1	1	0	0	0	0	0	1
		1/6/2019	0	0	0	1	1	1	1	0	1	1	0	1
006S	Age: 76M	19/10/2018	0	0	0	1	1	0	1	0	1	0	0	1
		22/05/2019	0	0	0	1	1	0	1	0	1	1	0	1
007S	Age: 81F	8/8/2018	0	0	0	0	1	0	0	0	1	1	0	1
		18/11/2019	0	0	0	0	1	0	1	0	1	0	0	0
008S	Age: 81M	4/5/2018	0	0	0	1	1	0	0	0	1	1	0	1
		29/09/2020	0	0	0	1	1	1	0	0	1	1	0	1
009S	Age: 83M	11/10/2018	0	0	0	0	1	0	0	0	1	1	0	1
		9/8/2019	0	0	0	0	1	0	1	0	1	1	0	0
010S	Age: 73F	27/04/2018	0	0	0	0	1	0	0	0	1	1	0	1
		3/8/2018	0	0	0	0	1	0	0	0	1	1	0	1
011S	Age: 87M	13/02/2019	0	0	0	0	1	0	0	0	1	1	0	1
		5/6/2019	0	0	0	0	1	0	0	0	1	1	0	1
012S	Age: 81M	16/11/2018	0	0	0	1	1	0	0	0	1	1	0	1
		6/5/2019	0	0	0	1	1	0	0	0	1	1	0	1
013S	Age: 84M	21/11/2018	0	0	0	1	1	0	0	0	1	1	0	1
		30/03/2019	0	0	0	1	1	0	0	0	1	1	0	1
014S	Age: 75M	5/6/2019	0	0	0	1	1	0	1	0	1	1	0	1
		3/2/2020	0	0	0	1	1	0	0	0	1	1	0	1
015S	Age: 82M	7/12/2019	0	0	0	1	1	1	1	0	1	1	0	1
		2/2/2020	0	0	0	1	1	0	0	0	1	1	0	1
016S	Age: 88M	23/08/2017	0	0	0	1	1	0	0	0	0	1	0	1
		9/12/2017	0	0	0	1	1	0	0	0	0	1	0	1
017S	Age: 91F	21/03/2021	0	0	0	0	1	0	0	0	1	1	0	1
		9/5/2021	0	0	0	1	1	0	0	0	1	1	0	0

**Table 3:** Individual Demographics and Behavioral Categories in Failed De-prescribing group. Columns show the individual patient’s demographics of identification number, age, and the initial and the second date of data collection. Rows show the individual behavioral categories with 0 representing the absence of the category and 1 representing the presence of the category.

Behavioural Categories														
Patient Number	Age	Date	Disorganized Expressions	Mis-Identification Expressions	Goal-Directed Expressions	Vocal Expressions	Emotional Expressions	Fretful-Trepida-ted Expressions	Importuning Expressions	Apathy Expressions	Oppositional Expressions	Physical Expressions	Sexual Expressions	Motor Expressions
001F	Age: 62 F	23/07/2017	0	1	1	1	1	1	1	0	1	1	0	1
		14/05/2016	0	0	0	0	1	1	1	0	1	1	0	1
002F	Age: 80 M	21/01/2019	0	0	0	0	1	1	1	0	1	1	0	1
		28/02/2019	0	1	1	1	1	1	1	0	1	1	0	1
003F	Age: 75M	2/12/2018	0	0	0	1	1	1	1	0	1	1	0	0
		25/09/2018	0	1	0	1	1	1	1	0	1	1	0	1
004F	Age: 84M	5/4/2019	0	0	0	0	1	0	0	0	1	1	0	0
		17/04/2019	0	1	1	1	0	0	0	0	1	1	0	1
005F	Age: 81 F	23/01/2019	0	0	0	1	1	0	0	1	1	1	0	1
		5/3/2019	0	1	1	1	1	0	0	1	1	1	0	1
006F	Age: 77M	27/02/2019	0	1	1	1	1	0	0	0	1	1	0	1
		3/7/2019	0	0	0	0	1	0	0	0	1	1	0	0
007F	Age: 98F	29/03/2019	0	0	0	0	1	1	1	0	1	0	0	1
		4/5/2019	0	1	1	0	1	1	1	0	1	0	0	1
008F	Age: 88 M	19/11/2018	0	0	1	1	1	0	0	0	1	1	1	1
		30/07/2020	0	0	0	0	1	0	0	0	1	1	1	0
009F	Age: 60 F	25/11/2015	0	0	0	0	1	1	1	0	1	0	0	1
		14/02/2016	0	1	1	1	1	0	1	0	1	1	0	1
010F	Age: 74M	5/2/2016	0	0	0	1	1	1	0	0	1	1	0	0
		5/5/2016	0	1	1	1	1	1	1	0	1	1	0	1
011F	Age: 72M	11/12/2018	0	0	0	1	1	1	0	0	1	1	0	0
		9/4/2019	0	0	1	1	1	1	0	0	1	1	0	1
012F	Age: 79F	28/05/2016	1	1	1	1	1	0	0	0	1	1	0	1
		31/08/2016	0	0	0	1	1	1	0	0	1	1	0	1
013F	Age: 87M	18/12/2017	0	0	0	1	1	0	0	0	1	1	0	1
		23/01/2018	0	1	1	1	1	0	0	0	1	1	0	1
014F	Age: 87F	14/11/2018	0	0	0	0	1	1	1	0	1	1	0	1
		21/11/2018	0	1	1	1	1	0	1	0	1	1	0	1
015F	Age: 88 M	17/01/2017	0	0	0	1	1	0	1	0	1	1	0	1
		21/07/2017	0	1	1	1	1	0	1	0	1	1	0	1
016F	Age: 77M	20/12/2018	0	0	0	0	1	0	1	0	1	1	0	1
		14/03/2019	0	1	1	1	1	0	1	0	1	1	0	1
017F	Age: 92F	25/04/2018	0	0	0	0	1	1	0	0	1	1	0	1
		25/07/2018	0	1	1	1	1	1	0	0	1	1	0	1
018F	Age: 88F	21/11/2017	0	0	0	1	1	1	1	0	1	1	0	1
		18/02/2018	0	1	1	1	1	1	1	1	1	1	0	1
019F	Age: 76F	5/1/2019	0	0	0	1	1	0	0	0	1	1	0	1
		3/10/2019	0	1	1	1	1	0	0	0	1	1	0	1
020F	Age: 81M	25/11/2019	0	0	0	1	1	1	1	0	1	1	0	1
		23/01/2020	0	1	1	1	1	0	1	0	1	1	0	1
021F	Age: 83F	23/01/2020	0	1	1	1	1	0	0	0	1	1	0	1
		9/2/2020	0	0	0	1	1	0	0	0	1	1	0	1
022F	Age: 92M	8/3/2020	0	0	0	1	1	0	1	0	1	1	1	0
		27/10/2020	0	1	1	1	0	0	0	0	1	1	0	1
023F	Age: 76M	26/05/2020	0	0	0	1	1	0	0	1	1	1	1	1
		30/07/2020	0	1	1	1	1	0	0	0	1	1	0	1

**Table 4:** Shows Behavioral Frequencies by Category and Assessment.

	Variables		Assessment #1	Assessment #2
Behavioural Category	Disorganized Expressions	Present	0	0
		Not Present	40	40
	Mis-Identification Expressions	Present	1	21
		Not Present	39	19
	Goal-Directed Expressions	Present	1	22
		Not Present	39	18
	Vocal Expressions	Present	24	35
		Not Present	16	5
	Emotional Expressions	Present	40	38
		Not Present	0	2
	Fretful-Trepidated Expressions	Present	16	14
		Not Present	24	26
	Importuning Expressions	Present	14	18
		Not Present	26	22
	Apathy Expressions	Present	2	1
		Not Present	38	39
	Oppositional Expressions	Present	39	40
		Not Present	4	0
	Physical Expressions	Present	36	37
		Not Present	4	3
	Sexual Expressions	Present	4	2
		Not Present	36	38
	Motor Expression	Present	33	37
		Not Present	7	3

**Table 5:** MANOVA-Cross-Factoring of Time and De-Prescribing Results with Behavior Score. * means statistical significance with either time or de-prescribing, not both. ** means statistical significance with both time and de-prescribing.

Independent Variables	Dependent Variables	F Value	*p*
Disorganized Expression	Time	-	-
	De-Prescription		
Mis Identification Expression	Time	136.56	<0.0001 **
	De-Prescription	100.94	<0.0001 **
Goal-Directed Expression	Time	219.00	<0.0001 **
	De-Prescription	161.87	<0.0001 **
Vocal Expression	Time	8.48	0.0047 *
	De-Prescription	2.05	0.1560
Emotional Expression	Time	-	-
	De-Prescription		
Fretful Trepidated Expression	Time	-	-
	De-Prescription		
Importuning Expression	Time	-	-
	De-Prescription		
Apathy Expression	Time	-	-
	De-Prescription		
Oppositional Expression	Time	-	-
	De-Prescription		
Physical Expression	Time	-	-
	De-Prescription		
Sexual Expression	Time	-	-
	De-Prescription		
Motor Expression	Time	1.94	0.1680
	De-Prescription	6.78	0.01107 *

**Table 6:** Chi-square Test of Relationship between Behavior Category and Successful De-Prescribing.

Variables			Successful% (N)	Failed% (N)	x^2^ (*p*)
Behavioral Category	Disorganized Expressions	Present	0.0 (0)	0.0 (0)	-
		Not Present	100 (17)	100 (23)	
	Mis-Identification Expressions	Present	0.0 (0)	91.30 (21)	29.119 (*p* < 0.0001)
		Not Present	100 (17)	8.70(2)	
	Goal-Directed Expressions	Present	0.0 (0)	95.62 (22)	32.374 (*p* < 0.0001)
		Not Present	100 (17)	4.348 (1)	
	Vocal Expressions	Present	76.47 (13)	95.62 (22)	1.7684 (*p* = 0.184)
		Not Present	23.53 (4)	4.348 (1)	
	Emotional Expressions	Present	100 (0))	91.30 (21)	0.2638 (*p* = 0.6075)
		Not Present	0 (17)	8.70 (2)	
	Fretful-Trepidated Expressions	Present	35.39 (6)	34.78 (8)	0.000 (*p* = 1)
		Not Present	64.71 (11)	65.22 (15)	
	Importuning Expressions	Present	41.18 (7)	47.88 (11)	0.0093 (*p* = 0.0237)
		Not Present	58.82 (10)	52.17 (12)	
	Apathy Expressions	Present	0.0 (0)	4.348 (1)	0.000 (*p* = 1)
		Not Present	100 (17)	95.62 (22)	
	Oppositional Expressions	Present	100 (17)	100(23)	-
		Not Present	0 (0)	0.0 (0)	
	Physical Expressions	Present	88.24 (15)	95.62 (22)	0.0746 (*p* = 0.7847)
		Not Present	11.76 (2)	4.348 (1)	
	Sexual Expressions	Present	5.88 (1)	4.348 (1)	0.000 (*p* = 1)
		Not Present	94.12 (16)	95.62 (22)	
	Motor Expression	Present	82.35 (14)	100 (23)	2.2129 (*p* = 0.1369)
		Not Present	17.65 (3)	0.0 (0)	

**Table 7:** Paired t-Test Confirmation of MANOVA with Cohen’s d Scores.

		n Behavior Present	Mean	Mean of the Differences	t	df	*p*	Cohen’s d
Disorganized Expressions	First Assessment	0		0	-	39	-	-
	Last Assessment	0						
Mis-Identification Expressions	First Assessment	1	0.025	−0.5	−6.245	39	<0.0001	−0.9874
	Last Assessment	**21**	**0.525**					
Goal-Directed Expressions	First Assessment	1	0.025	−0.525	−6.566	39	<0.0001	−1.0381
	Last Assessment	22	0.55					
Vocal Expressions	First Assessment	24	0.6	−0.275	−3.846	39	0.0004	−0.6081
	Last Assessment	35	0.875					
Emotional Expressions	First Assessment	40	1	0.05	1.4327	39	0.1599	0.2265
	Last Assessment	38	0.95					
Fretful-Trepidated Expressions	First Assessment	16	0.4	0.05	0.7026	39	0.4865	0.1111
	Last Assessment	14	0.35					
Importuning Expressions	First Assessment	14	0.4	−0.05	−0.703	39	0.4865	−0.1111
	Last Assessment	18	0.45					
Apathy Expressions	First Assessment	2	0.05	0.025	1	39	0.3235	0.1581
	Last Assessment	1	0.025					
Oppositional Expressions	First Assessment	39	0.975	−0.025	−1	39	0.3235	−0.1581
	Last Assessment	40	1					
Physical Expressions	First Assessment	36	0.9	−0.025	−0.573	39	0.5703	−0.0905
	Last Assessment	37	0.925					
Sexual Expressions	First Assessment	4	0.1	0.05	1.4327	39	0.1599	0.2265
	Last Assessment	2	0.05					
Motor Expression	First Assessment	33	0.825	−0.1	−1.275	39	0.2099	−0.2016
	Last Assessment	37	0.925					

## Data Availability

3rd Party Data. Restrictions apply to the availability of these data. Data was obtained from Homewood Health Inc. and may be available through Shannon Remers (sremers@homewoodhealth.com) with the permission of Homewood Health Inc (Guelph, ON, Canada).
